# Impact of the new ultra-high sensitivity mode in a long axial field-of-view PET/CT

**DOI:** 10.1007/s12149-023-01827-y

**Published:** 2023-03-13

**Authors:** Clemens Mingels, Sabine Weidner, Hasan Sari, Dorothee Buesser, Konstantinos Zeimpekis, Kuangyu Shi, Ian Alberts, Axel Rominger

**Affiliations:** 1 Department of Nuclear Medicine, Inselspital, Bern University Hospital, University of Bern, Street: Freiburgstr. 18, 3010 Bern, Switzerland; 2 Advanced Clinical Imaging Technology, Siemens Healthcare AG, Lausanne, Switzerland

**Keywords:** Whole-body PET/CT, LAFOV PET/CT, Ultra-high sensitivity, High sensitivity, Acquisition time

## Abstract

**Objective:**

Long axial field-of-view (LAFOV) PET/CT showed improved performance resulting from higher sensitivity. The aim was to quantify the impact of using the full acceptance angle (UHS) in image reconstructions with the Biograph Vision Quadra LAFOV PET/CT (Siemens Healthineers) compared to the limited acceptance angle (high sensitivity mode, HS).

**Methods:**

38 oncological patients examined on a LAFOV Biograph Vision Quadra PET/CT were analysed. 15 patients underwent [^18^F]FDG-PET/CT, 15 patients underwent [^18^F]PSMA-1007 PET/CT, and 8 patients underwent [^68^Ga]Ga-DOTA-TOC PET/CT. Signal-to-noise ratio (SNR) and standardised uptake values (SUV_mean/max/peak_) were used to compare UHS and HS with different acquisition times.

**Results:**

The SNR was significantly higher for UHS compared to HS over all acquisition times (SNR UHS/HS [^18^F]FDG: 1.35 ± 0.02, *p* < 0.001; [^18^F]PSMA-1007: 1.25 ± 0.02, *p* < 0.001; [^68^Ga]Ga-DOTA-TOC: 1.29 ± 0.02, *p* < 0.001).

**Conclusion:**

UHS showed significantly higher SNR opening the possibility of halving short acquisition times. This is of advantage in further reduction of whole-body PET/CT acquisition.

## Introduction

Nuclear medicine techniques especially in hybrid imaging have undergone rapid development since the first introduction of a clinical positron emission tomography/computed tomography (PET/CT) system [1]. The recently introduced LAFOV scanners in Philadelphia, Pennsylvania, USA (PennPET Explorer), in Davis, California, USA (uExplorer, United Imaging Healthcare America) and in Bern, Switzerland (Biograph Vision Quadra, Siemens Healthineers) demonstrated that the longer coverage of coincident photons results in higher count density and higher sensitivity of the LAFOV PET/CT systems. Accordingly, signal-to-noise ratio (SNR) of the scanner increases with longer FOV (2).

Recent work has shown that scan time can be reduced with a LAFOV PET system [3, 4]. Yet, at the time of the first publication of the latest LAFOV Biograph Vision Quadra PET/CT system, image reconstructions using the full acceptance angle (maximum ring difference (MRD) 322) were not available and reconstruction was restricted to a limited acceptance angle (MRD 85) [5, 6]. Detecting coincidences with full acceptance angle is known to be challenging in LAFOV system. Zhang et al. observed in the uEXPLORER total-body PET-scanner worsening axial resolution with increasing MRD since oblique lines-of-response (LOR) are more likely to be scattered in the patient with longer MRD [7]. Therefore, fast time-of-flight (TOF) resolution and accurate scatter correction are crucial to avoid misinterpretation of the coincident events [8, 9].

Here, we present the first clinical data with the new ultra-high sensitivity (UHS) reconstruction mode (MRD 322) in the Biograph Vision Quadra and aim to evaluate it with regard to noise rate, lesion quantification and acquisition time.

## Materials and methods

### Patient population

**Fig. 1:**
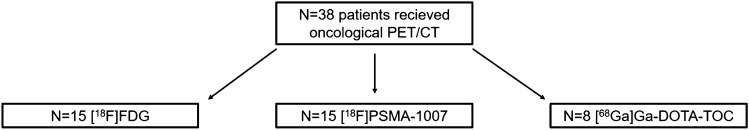
Study flowchart showing patient recruitment and the patients who were included

38 previously published patients undergoing clinically routine oncological PET/CT between October 2020 and December 2020 were analysed retrospectively [5]. Data from 15 patients receiving a [^18^F]FDG-PET/CT, 15 patients receiving [^18^F]PSMA-1007 PET/CT and 8 patients receiving [^68^ Ga]Ga-DOTA-TOC PET/CT were analysed (Fig. 1) [10]. 10 min list-mode (LM) data acquisition was utilised for all scans.

### Imaging protocol

The PET LM data were calculated using 600, 360, 240, 120, 60, 30, 20, 10, 5 and 2 s durations to simulate shorter acquisition times. Long acquisition times were considered as > 120 s, short acquisitions as 120–30 s and very short acquisitions as < 30 s. Biograph Vision Quadra acquires the PET emission data using the maximum full ring difference (MRD 322), and offers two reconstruction modes: ultra-high sensitivity mode (UHS) with the full ring difference and full acceptance angle (54 degrees) and high sensitivity (HS) mode with limited ring difference (MRD 85) and acceptance angle (19 degrees) [11, 12]. In this work, we reconstructed the PET data with UHS and HS modes. Image reconstruction was performed using a dedicated image reconstruction software (e7-tools, Siemens Healthineers), which was available at our clinic at the time of analysis and will be provided by Siemens Healthineers to general users in the future. In all reconstructions, point-spread-function (PSF)-TOF method was used with four iterations and five subsets and were reconstructed with a 440 × 440 × 644 image matrix with a voxel size of 1.65 × 1.65 × 1.65 mm^3^. A Gaussian post-reconstruction filter with 2 mm full width at half maximum (FWHM) was applied to the images. Attenuation correction was performed using the low-dose non-enhanced CT data. 3D scatter correction was performed using a 3D residual-based method which was shown to be more accurate than 2D single scatter simulation method with large acceptance angles and oblique lines-of-response (i.e. MRD 322) [13]. All images in both MRD 85 and 322 were reconstructed using the 3D scatter correction.

### Image evaluation

Image analysis and identification of target lesions (malignant tissue) were performed using a separate software (pmod; PMOD Technologies LLC, Zürich, Switzerland). Lesion uptake and metabolic tumour volumes were calculated by placing a volume-of-interest (VOI) with a 40%-iso-contour approach around the lesion as previously described [5, 14].

Peak and maximum standardised uptake values (SUV_peak/max_) were used to evaluate target lesion knowing that SUV_peak_ has been shown to be less susceptible for variation at different acquisition times than SUV_max_ [15].

The background was measured by the placement of a 10 cm^3^ VOI in healthy liver tissue in the right liver lobe as previously described [16]. Using the PMOD software, VOIs were automatically applied to all different images obtained from different frame durations. The SNR was defined as the reciprocal coefficient of variation (COV) for the liver background, where σ was the standard deviation of the background VOI and μ was the SUV_mean_ of the background VOI [5].

### Statistical analysis

Statistical analyses were performed using Graphpad Prism Version 9 (San Diego, California). The data are presented either as mean ± standard deviation (SD) or as median ± standard error (SE). Comparison between the different SNR and SUV measurements were characterised using paired Student`s *T* test and applying Bonferroni correction. *p* values less than 0.0011 were considered statistically significant (marked with an asterisk “*”) according to Bonferroni correction.

## Results

### Patient examination

In total, 153 target lesions were identified in 38 patients in the reference standard of 10 min acquisition time. Patients’ characteristics including the tumour type, mean administered radiopharmaceutical activity ± standard deviation, and age are outlined in Table 1.

**Table 1:** Patients’ characteristics of the included patients (*n* = 38). Given are radiopharmaceuticals, tumour types, mean and SD of the activity (MBq) and age (mean)

Radiopharmaceutical	Tumour type	Activity (MBq)	Age (years)
[^18^F]FDG	Lung: 6, lymphoma: 4, ORL: 3, breast: 2	265.6 ± 65.8	67.9
[^18^F]PSMA-1007	Biochemical recurrence: 13, primary: 2	243.9 ± 14.0	75.5
[^68^ Ga]Ga-DOTA-TOC	SSTR-expressing neuroendocrine tumours: 8	154.1 ± 12.0	65.3

### Signal-to-noise ratio (SNR) in three different radiopharmaceuticals from 2 to 600 s

**Fig. 2:**
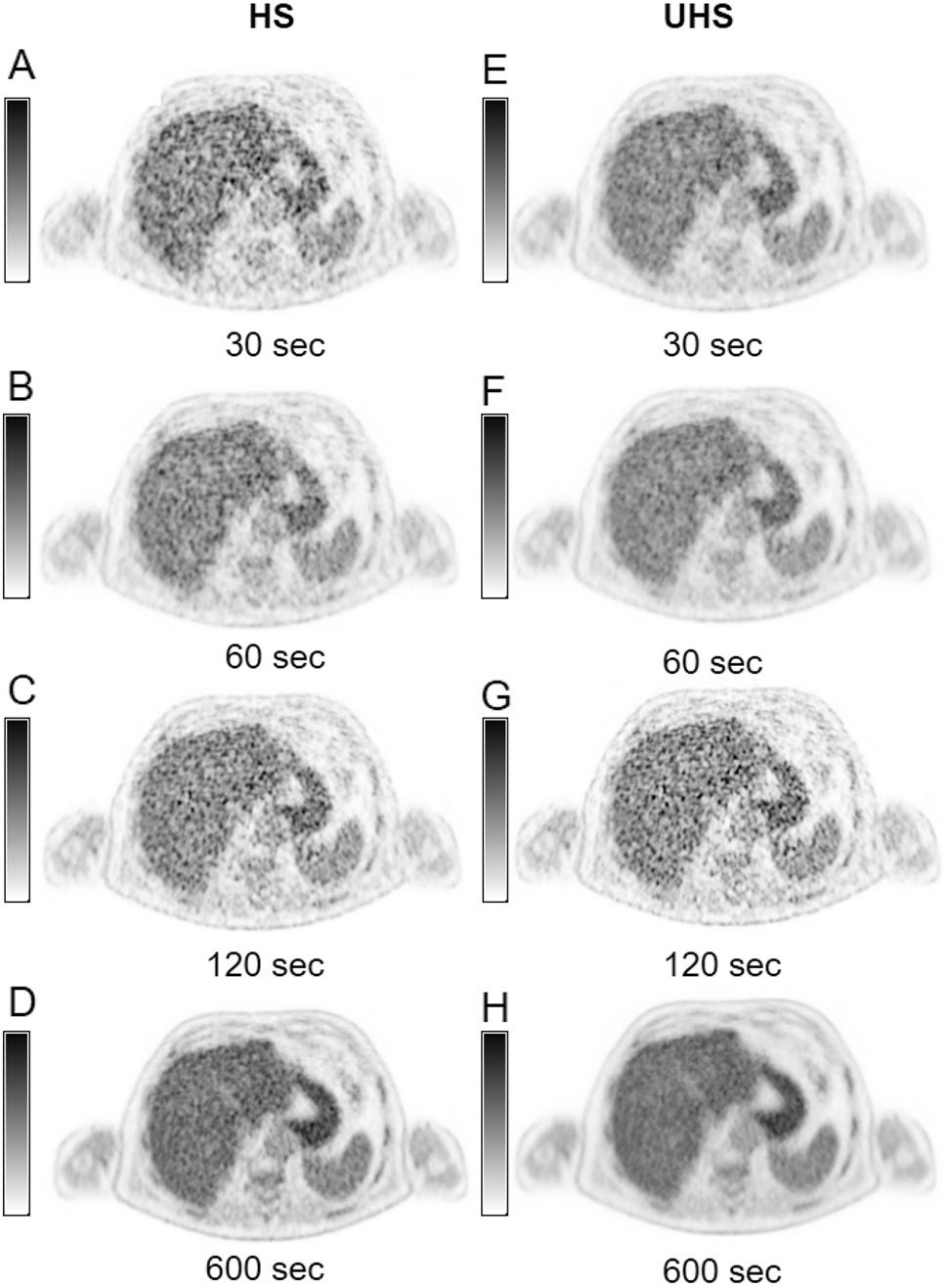
Example images of liver signal (SUV_max_) in both high sensitivity and ultra-high sensitivity reconstructions (HS: **A**–**D**; UHS: **E**–**H**) in different acquisition times (30 s, 60 s, 120 s, 600 s)

**Fig. 3:**
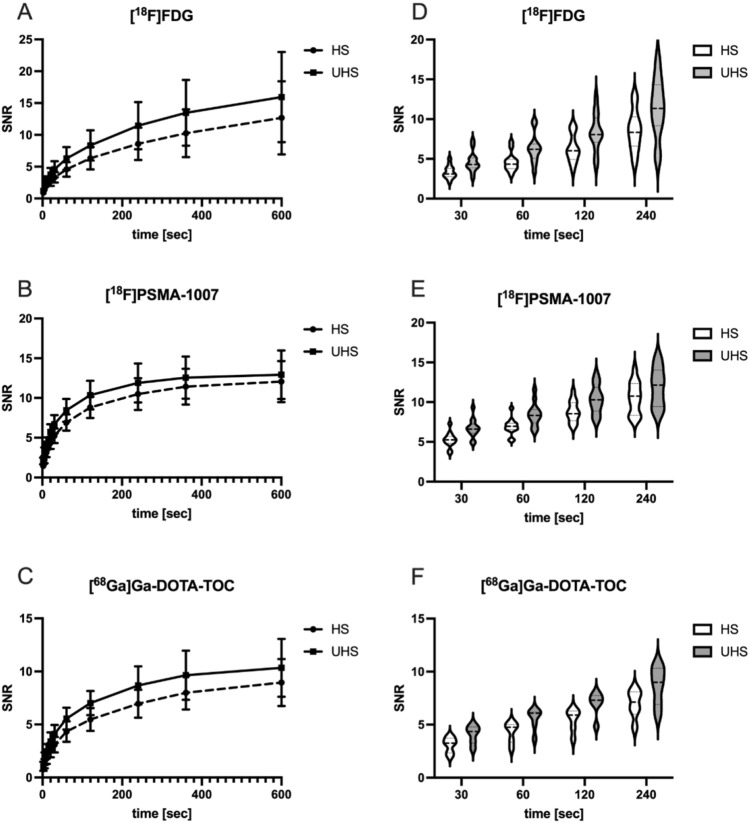
Signal-to-noise ratios (SNR) of all three radiopharmaceuticals in high sensitivity (HS) and ultra-high sensitivity reconstruction (UHS) as reciprocal coefficient of the variance (COV). Given are the SNR in all three radiopharmaceuticals for 2 s, 5 s, 10 s, 20 s, 30 s, 60 s, 120 s, 240 s, 360 s and 600 s acquisition time (**A**–**C**). All SNR of UHS were significantly higher to HS mode. Shown are mean ± SD. Comparable SNR could be found between 30 s UHS and 60 s HS, between 60 s UHS and 120 s HS and between 120 s UHS and 240 s HS in all three radiopharmaceuticals as visualised in the violin plots (**D**–**F**)

We report significantly higher SNR in all three examined radiopharmaceuticals for UHS compared to HS reconstructions (SNR UHS/HS ratio: [^18^F]FDG: 1.35 ± 0.02, *p* < 0.001; [^18^F]PSMA-1007: 1.25 ± 0.02 *p* < 0.001; [^68^ Ga]Ga-DOTA-TOC: 1.29 ± 0.02, *p* < 0.001) over all acquisition times. Figure 2 shows examples of the reconstructed images in both HS and UHS with various acquisition times. As supported by the visual impression (example images Fig. 2), liver signal increased with increasing acquisition time in all three radiopharmaceuticals (Fig. 3 A–C). Furthermore, the SNR in HS short acquisition time was comparable with half of its time counterpart in UHS ([^18^F]FDG: HS 60 s: 4.58 ± 1.12 vs. UHS 30 s: 4.60 ± 1.24; HS 120 s: 6.32 ± 1.68 vs. UHS 60 s: 6.27 ± 1.72 and HS 240 s: 8.59 ± 2.345 vs. UHS 120 s: 8.34 ± 2.28). Detailed information of all three radiopharmaceuticals can be found in Fig. 3 D–F.

### Target lesion values represented by SUV_peak_ and SUV_max_

**Fig. 4:**
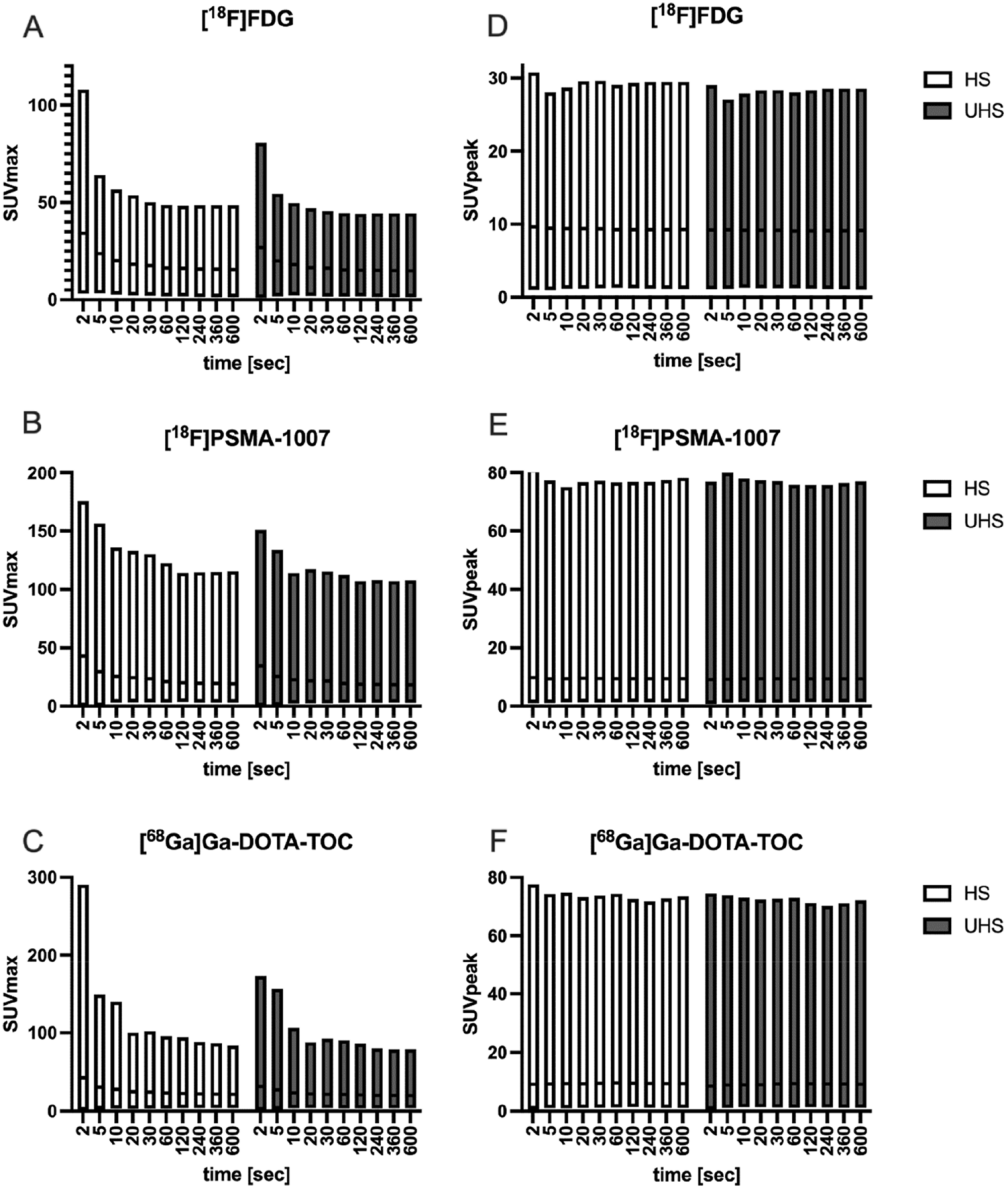
Median ± standard error of standardised uptake values (SUV_max_ and SUV_peak_) for tumour lesions in [^18^F]FDG (**A**, **D**), [^18^F]PSMA-1007 (**B**, **E**) and [^68^ Ga]Ga-DOTA-TOC (**C**, **F**) in high sensitivity (HS) and ultra-high sensitivity (UHS) mode

SUV_peak_ in HS was 5.34 ± 1.04 for [^18^F]FDG and 5.48 ± 1.02 in UHS over all acquisition times. SUV_peak_ was constant over all acquisition times and there was no evidence of statistical significant difference between UHS and HS. Median ± standard error SUV_max_ was 13.63 ± 1.84 in HS and 12.14 ± 1.62 in UHS. No evidence of statistical significant differences between HS and UHS SUV_max_ could be found. For [^18^F]PSMA-1007, median ± standard error SUV_peak_ in HS was 5.39 ± 1.23 and in UHS was 5.25 ± 1.22 over all acquisition times. SUV_max_ was 16.72 ± 3.20 in HS and 15.33 ± 2.87 in UHS. No statistical significant differences between HS and UHS SUV_peak/max_ could be seen. Median ± standard error SUV_peak_ for the patients with [^68^ Ga]Ga-DOTA-TOC in HS was 4.73 ± 1.2 and 4.74 ± 1.19 in UHS over all acquisition times. SUV_max_ was 14.05 ± 3.39 in HS and 12.68 ± 2.96 in UHS. No statistical significant differences between HS and UHS SUV_peak/max_ could be seen. Details of all SUV_max/peak_ at all acquisition times are outlined in Fig. 4.

## Discussion

In this study, we show the first in-human evaluation of the ultra-high sensitivity mode (MRD 322) of the Biograph Vision Quadra PET/CT system. Previously published studies with the Biograph Vision Quadra were limited to a MRD of 85 crystal rings which limited the acceptance angle [5, 11, 17]. Using the full acceptance angle to detect coincidences (MRD 322), the NEMA sensitivity of the Biograph Vision Quadra PET/CT increased in phantom studies by the factor of two compared to MRD 85 (sensitivity MRD 85: 83.4 cps/kBq vs. MRD 322: 176 cps/kBq) [11].

In our clinical analysis, we report significant increase of the SNR as an indicator for the higher sensitivity of the scanner in UHS mode (MRD 322) compared to HS mode (MRD 85). We examined the SNR in the liver of patients receiving either [^18^F]FDG, [^18^F]PSMA-1007 or [^68^ Ga]Ga-DOTA-TOC. In all three radiopharmaceuticals, SNR was significantly higher in UHS compared to HS mode. By increasing acquisition time, SNR also increased (Fig. 3 A–C). SNR of 30 s/60 s acquisition in UHS was comparable to 60 s/120 s in HS mode (Fig. 3 D–F).Visual impression (Fig. 2) supported those findings.

Individual standardised uptake values (SUV_peak/max_) for all 153 tumour lesions were similar between the three radiopharmaceuticals over different acquisition times. Comparable SUV_peak/mean_ even in short acquisitions (30–120 s) and high SUV_max_ (up to 20% higher) in short acquisitions are already described due to higher noise rates [18–20].

Accordingly, in our study, SUV_peak_ was stable from 2 s acquisition up to 600 s acquisition in both reconstruction modes and SUV_max_ was high in the very short acquisitions (ca. 100% higher than after 60 s at 2 s of acquisition). Using UHS in short acquisition times (30–120 s) lead to reduced bias of SUV_max_. After 30 s of scanning, SUV_max_ was comparable to the longer acquisitions in UHS whereas, in HS, comparable SUV_max_ could be found after 60 s of scanning ([^18^F]FDG: UHS 30 s: 12.27 ± 1.55, HS 120 s: 12.15 ± 1.52), only. The risk of misinterpreting the semi-quantitative SUV_max_ measurements in short acquisition times is a problem, which can be partially addressed using the UHS mode.

We note some limitations. UHS’s sensitivity profile is non-uniform, unlike in HS. Sensitivity in MRD 85 is uniform up to the very last centimetres at the scanner whereas MRD 322’s point of highest sensitivity is in the middle of the scanner and sensitivity decreases to the edges [11]. SNR might, therefore, differ in UHS when examining lesions at the edge of the scanner (e.g. brain or skin lesions at the lower extremities). Further experiments, especially phantom studies might be able to characterise the absolute Bq/ml in UHS and HS. This is not accounted for in our study where the analysis was limited to the liver, which is placed in the middle of the LAVOV PET system and will be improved with the availability of continuous bed motion.

## Conclusion

Using the UHS mode (MRD 322) on the LAFOV PET/CT Biograph Vision Quadra might be of clinical advantage due to significantly higher SNR relative to the HS mode (MRD 85) in all examined radiopharmaceuticals. This opens the possibility of halving the acquisition time using UHS in short PET acquisitions, leading to an improvement in clinical management of oncological patients.

## Data Availability

All data are available and can be found at the corresponding author’s address.
